# Analysis of Codon Usage of Speech Gene *FoxP2* among Animals

**DOI:** 10.3390/biology10111078

**Published:** 2021-10-21

**Authors:** Tarikul Huda Mazumder, Ali M. Alqahtani, Taha Alqahtani, Talha Bin Emran, Afaf A. Aldahish, Arif Uddin

**Affiliations:** 1 EduCare Academy, Silchar 788006, Assam, India; tariqulhmazumder@gmail.com; 2 Department of Pharmacology, College of Pharmacy, King Khalid University, Abha 62529, Saudi Arabia; amsfr@kku.edu.sa (A.M.A.); ttaha@kku.edu.sa (T.A.); adahesh@kku.edu.sa (A.A.A.); 3 Department of Pharmacy, BGC Trust University Bangladesh, Chittagong 4381, Bangladesh; talhabmb@bgctub.ac.bd; 4 Department of Zoology, Moinul Hoque Choudhury Memorial College, Hailakandi 788150, Assam, India

**Keywords:** *FoxP2* gene, effective number of codons, relative synonymous codon usage, natural selection

## Abstract

**Simple Summary:**

We evaluated codon usage bias in the *FoxP2* gene in fishes, birds, reptiles, and mammals. Fishes use C or G—ending codons, while birds, reptiles, and mammals employ T or A—ending codons. Apart from the nucleotide composition, natural selection and mutation pressure might influence the CUB. The ENC observed/ENC expected ratio demonstrated that mutation pressure influences *FoxP2* codon usage patterns. Natural selection may have had a key influence in shaping the CUB, although mutation pressure may have played a minor role. *FoxP2* gene codon usage is affected by the base composition under mutation bias.

**Abstract:**

The protein-coding gene *FoxP2* (fork head box protein P2) plays a major role in communication and evolutionary changes. The present study carried out a comprehensive codon usage bias analysis in the *FoxP2* gene among a diverse group of animals including fishes, birds, reptiles, and mammals. We observed that in the genome of fishes for the *FoxP2* gene, codons ending with C or G were most frequently used, while in birds, reptiles, and mammals, codons ending with T or A were most frequently used. A higher ENC value was observed for the *FoxP2* gene indicating a lower CUB. Parity role two-bias plots suggested that apart from mutation pressure, other factors such as natural selection might have influenced the CUB. The frequency distribution of the ENC observed and ENC expected ratio revealed that mutation pressure plays a key role in the patterns of codon usage of *FoxP2*. Besides, correspondence analysis exposed the composition of the nucleobase under mutation bias affects the codon usage of the *FoxP2* gene. However, neutrality plots revealed the major role of natural selection over mutation pressure in the CUB of *FoxP2*. In addition, the codon usage patterns for FoxP2 among the selected genomes suggested that nature has favored nearly all the synonymous codons for encoding the corresponding amino acid. The uniform usage of 12 synonymous codons for FoxP2 was observed among the species of birds. The amino acid usage frequency for FoxP2 revealed that the amino acids Leucine, Glutamine, and Serine were predominant over other amino acids among all the species of fishes, birds, reptiles, and mammals.

## 1. Introduction

The standard genetic code makes use of 64 codons to characterize the 20 standard amino acids, including 3 stop codons, during the translation of a protein. The redundancy of the genetic code implies that one amino acid may be encoded by more than one codon (except methionine and tryptophan) leading to the occurrence of synonymous codon usage bias (CUB) in the genome of an organism, which varies among diverse groups [1]. Codon usage analyses have displayed that codon utilization bias is very complex and is accompanied by numerous organic influences, which include gene expression [2,3,4], gene length, the gene translation initiation sign [5], nucleotide composition [6], protein amino acid composition [7,8], protein structure [9,10], tRNA abundance [11,12,13,14], mutation frequency and patterns [15,16], environmental temperature [17], GC composition [18,19,20,21], and the balance between natural selection and gene mutation [22]. Hence, analyses of CUB are important in understanding the molecular evolution of a gene or genome of an organism, adaptation to different environmental conditions, genomic architecture, and the prediction of related functional gene expression.

*FoxP2* was reported in 1990 as a putative “speech gene” that binds to the fork head box protein P2 and the autosomal dominant trait, causing a severe and specific speech disorder [23]. These findings attracted the scientific community to study the FoxP2 phenotype in diverse taxa including mammals, birds, and insects [24]. Earlier studies identified that the expression of the *FoxP2* gene was linked to several brain regions for vocal development in primates [25], rodents [26], and songbirds [27]. Moreover, the protein-coding sequence of FoxP2 is amongst the most extremely conserved 5% of proteins in vertebrates and plays a key role in modulating vocal learning and communication, which might be shared by a range of animal species [28,29,30]. Recently, it was reported that the protein sequence of zebra finch FoxP2 is 98% identical to that of mouse and human FOXP2 [31]. Songbirds are an appropriate model for researching the mechanisms of imitative vocal learning, as well as speech and its pathologies. The expression patterns of *FoxP2* in songbird and human brains are identical, with strong expression in the basal ganglia, thalamus, and cerebellum [31,32]. However, studies of FoxP2 are limited to nonvocal/nonsonic species of teleost fish such as medaka (*Orizya latipes*), zebrafish (*Danio rerio*), and others [33,34].

Here, in this study, we have tried to elucidate the differences in the nucleotide coding sequence of the evolutionary conserved FoxP2 gene among various species of teleost fish, birds, reptiles, and mammals. This is the first comprehensive study of CUB in four groups of the *FoxP2* gene that thoroughly described the role of evolutionary forces and the evolutionary genetic relationship in these specific genes.

## 2. Materials and Methods

The complete nucleotide coding sequence (CDS), along with the accession number, of the *FoxP2* gene among different species of fishes, birds, reptiles, and mammals has been retrieved from GenBank of the NCBI database (Table S1). The CDS, which has a perfect start codon as well as a stop codon and no unknown nucleobase in the middle of the sequence, was used in our CUB analysis. 

### 2.1. Codon Usage Bias and Codon Usage Indices

The GC3 value is an important parameter to measure the degree of nucleobase composition bias since the proportion of the nucleobase GC contents varies at their third position of a synonymous codon [35]. 

Moreover, GC contents at the different codon positions of P1, P2, P3, and P12 were calculated. The P12 value represents the average value of P1 and P2 and is generally used to perform a neutrality plot analysis.

The Effective Number of Codons (ENC) values are used to measure the CUB of a gene, and its value ranges from 20 to 61 [36]. A greater ENC value represents weak codon bias, indicating the synonymous codon is used equally to code for the corresponding amino acid. The expected ENC value is calculated using the formula.

(1) $$ENC^{expected}=2+s+\frac{29}{s^{2}+\left(1-s^{2}\right)}$$

where *s* is the frequency of GC3 [36].

Further, the frequency distribution of observed ENC and expected ENC [(ENC exp-ENC obs)/ENC exp] is calculated and plotted.

### 2.2. Relative Synonymous Codon Usage

The relative synonymous codon usage (RSCU) is computed by dividing the observed frequency of a codon by the expected frequency when there is uniform usage of the synonymous codon [4]. An RSCU value greater than 1.0 or less than 1.0 represent the more frequently (preferred) and less frequently (rare) used codons than expected, respectively, while an RSCU value greater than 1.6 indicates an over-represented codon for encoding the particular amino acid [4,37].

### 2.3. PR2 Plot

The parity rule plots i constructed by utilizing the values of both GC bias [G3/(G3 + C3)] and AT bias [A3/(A3 + T3)] and is used to determine the effect of mutation and selection pressure [38]. The center of the plot is 0.5 and represents no bias for selection and mutation in the two complementary strands of DNA. 

### 2.4. Correspondence Analysis

Correspondence analysis is a multivariate statistical tool that is generally used to determine the most important trends in the codon usage variation among the genes and distribute the codons in *axis1* and *axis2* [39,40]. Hence, to explore the variation in the codon usage of the *FoxP2* gene within the species of different groups, a correspondence analysis was performed based on *RSCU* values. 

### 2.5. Neutrality Plot

A neutrality plot is generally used to determine the factor affecting the patterns of codon usage and codon bias along with the characterization of the relationship between GC3 and GC12. In this graphical plot, the regression with a slope close to 0 represents no effect of directional mutation pressure (a dominant role of natural selection) while a slope close to 1 represents complete neutrality (a dominant role of mutation pressure) [41].

### 2.6. Software Used

The ENC values of each CDS for the *FoxP2* gene of the selected species among fish, birds, reptiles, and mammals were calculated using published online software from the Computational Biology and Bioinformatics Lab, Tezpur University, Assam, India [42]. The nucleotide composition, codon usage, amino acid usage, and phylogenetic analyses were performed using Mega 6.0 [43]. A heat map of correlation analyses between codon usage and GC3 values was produced in the online Heatmapper software [44]. Correspondence analysis was conducted using past software [45]. GRAVY and AROMATICITY was calculated using Galaxy [46]. 

### 2.7. Statistical Analysis

All statistical calculations including correlation analysis between codon usage and indices were performed in IBM SPSS version 21.0. The figures were evaluated using Microsoft Excel 16.0.

## 3. Results

### 3.1. Indices of Codon Usage

The mean length of the CDs, the composition of the GC contents along with its different codon positions (Length_cds_, GC_cds_, P1, P2, P3, P12), and the codon usage indices such as ENC, Aromaticity, and GRAVY among fishes, birds, reptiles, and mammals are listed in Table 1.

The ENC value reflects the extent of codon bias in a gene. In our study, we observed a mean value of ENC for the *FoxP2* gene among fishes, reptiles, birds, and mammals greater than 50 (Table 1), suggesting low codon bias exists among these organisms.

**Figure 1:**
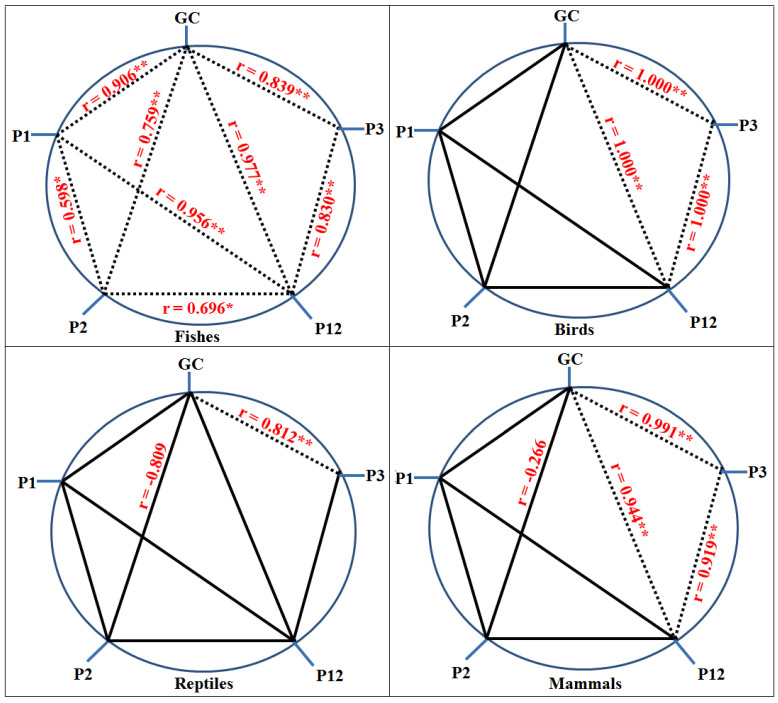
The correlation analysis between the mean value of GC contents and its different codon positions P1, P2, P3, and P12 for *Foxp2* genes among fishes, birds, reptiles, and mammals. The dotted lines with asterisk (*) represents significant correlation while the line without dots represents no significant correlation detected in their Pairwise analysis. * *p* < 0.05 and ** *p* < 0.01.

The composition of the nucleotides is another essential factor that affects the CUB. The nucleotide composition and ENC value along with the mean and standard deviation in the CDS of the *FoxP2* gene among different species of fish, birds, reptiles, and mammals were calculated (Table S2). The overall nucleotide composition analysis in fishes showed that the percentage of nucleobase C was the highest, followed by A, G, and T, while for the *FoxP2* gene in birds, reptiles, and mammals, nucleobase A was the highest followed by C, G, and T. Similarly, the nucleobase at the third codon position in fishes and mammals showed that G3% was the highest followed by C3%, A3%, and T3%, whereas in birds and reptiles, A3% was the highest followed by G3%, T3%, and C3% (Table 2). The overall percentage of AT composition in comparison to GC composition was higher in birds, reptiles, and mammals while in fishes, the overall percentage of GC composition was higher than the AT composition. In addition, P3 content was also highest in fishes in comparison to reptiles, birds, and mammals for the *FoxP2* gene (Table S2). The mean percentage of GC composition for the *FoxP2* gene among the selected organisms ranged from 46.8% to 55.8%. However, the GC composition mean percentage at positions P1, P2, P3, and P12 (the average of P1 and P2) was significantly different among different organisms. The analysis of the correlation coefficients (Figure 1) showed that the mean value of GC content was significantly correlated at positions P3 and P12 for the *FoxP2* gene among all the organisms except reptiles, which indicates that codon usage in the CDS of the *FoxP2* were affected by the general GC contents of the organisms [47]. In fishes, P12 and both P1 and P2 showed a strong positive correlation (*p* < 0.01), indicating the influence of mutation pressure, but no significant correlation was detected in birds, reptiles, and mammals (Figure 1), which indicates the influence of natural selection over mutation pressure in the CUB of *FoxP2* among these organisms [41]. However, the correlation between ENC and P3 showed a significant negative correlation (r = −0.938, *p* < 0.01) in fishes and a significant positive correlation (r = 1.000, *p* < 0.01) in mammals, as well as no significant correlation in birds and reptiles (Table 3). The above results indicated that the relationship of GC3 values with ENC values in fishes and mammals representing the mutation pressure accounted for the patterns of the nucleobase composition bias [48]. Earlier it was reported that the extent of P3 distribution may be linked to the divergence of directional selection and that of mutation pressure [49]. In our analysis, the P3 values for the *FoxP2* gene among different groups were distributed between 0.4 and 0.7, suggesting that the *FoxP2* gene in diverse groups mainly evolved via mutation pressure [50].

**Figure 2:**
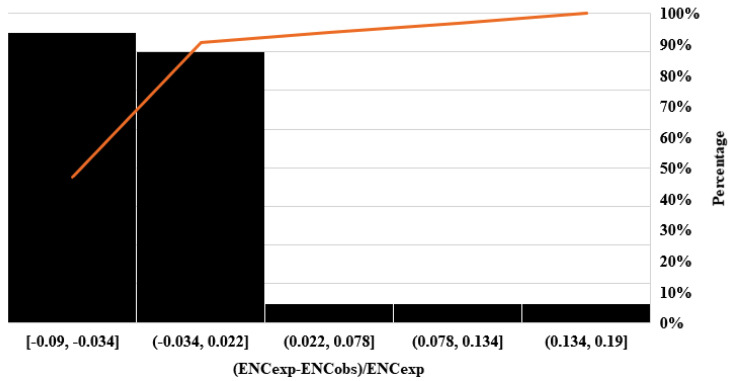
The frequency distribution plot for effective number of codons ratio of observed ENC and expected ENC [*(ENCexp-ENCobs)/ENCexp*].

Moreover, the difference in ENC values between the observed and expected values was calculated using the formula [(ENCexp-ENCobs)/ENCexp] for all CDS of the *FoxP2* gene among diverse groups and plotted to detect the frequency of variation (Figure 2). Nearly all the CDS of *FoxP2* genes emerged in the range of −0.9 to −0.03 and −0.03 to 0.02, which indicate that most of the ENC observed values are smaller than that of the ENC expected values. Thus, the result revealed that the codon usage of the *FoxP2* gene can be ascertained based on GC3 values and mutation pressure, which play a key role in the patterns of codon usage [50].

In addition, we performed correlation analysis between the overall composition of the nucleotide (A%, T%, G%, C%, GC%) and its codon in the third position (A3%, T3%, G3%, C3%_,_ GC3%) (Table 2) to detect the effects of translational selection or mutational pressure on the codon bias of the *FoxP2* gene among diverse groups. We observed a significant correlation with a positive value among homogeneous nucleotides and a significant correlation with a negative value among most of the heterogeneous nucleotides, which suggested that mutational pressure affects the base composition bias of the *FoxP2* gene [37].

### 3.2. Codon Usage Pattern

**Figure 3:**
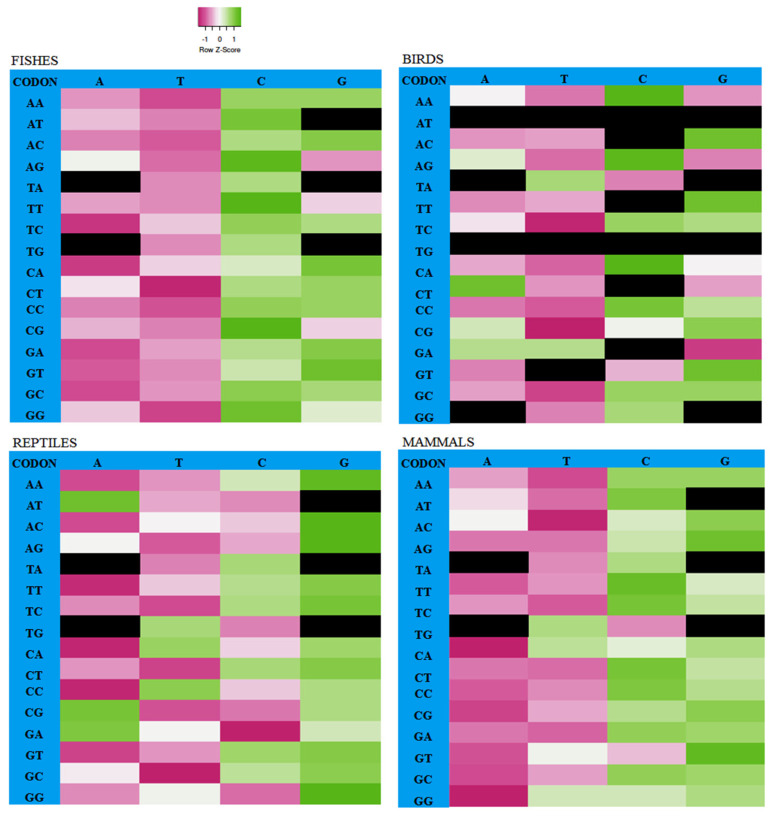
The correlation analysis between synonymous codon usage and GC3 values using a heatmap. The black fields represent stop codons (TAA, TAG, and TGA) and non-degenerate codons (ATG, TGG). Uniform usage codons (ATA, ATT, ATC, ACC, TTC, TGT, TGC, CTC, GAC, GTT, GGA, and GGG) were observed only among bird species and are represented as black color.

The correlation between synonymous codon usage and GC3 values in the CDS of the *FoxP2* gene among the studied organisms (Figure 3) showed that nearly all AT-ending codons were negatively correlated with GC3s while GC-ending codons were positively correlated with GC3s, which indicates that the frequency of synonymous codon usage depends on the increased bias of GC contents [51]. We observed that nearly all the synonymous codons were randomly used for encoding the corresponding amino acids among all the species of the selected genome for the *FoxP2* gene. In the case of the bird genome, we observed 12 synonymous codons that were uniformly used among different species of birds for the *FoxP2* gene (Figure 3), suggesting nature maintains its functional property throughout the period of evolution [52].

Analysis of overall non-uniform usage of synonymous codons i.e., the RSCU values for the *FoxP2* gene of different species in each of the studied organisms were calculated (Table S3). In our analysis, the more frequently used codons (RSCU > 1.0) in fishes amounted to 23, including C-12, G-5, A-4, and T-2 as well as the over-represented codons (RSCU > 1.6, marked as yellow in Table S2) of which there were 6, namely AGA (R), CAG (Q), CGC (G), ATC (Ile), CTG (L), AGC (S), and GTG (G). Similarly, in birds, the most frequently used codons amounted to 25, including T-11, A-9, G-3, and C-2 as well as 6 over-represented codons, namely GCA (A), AGA (R), GGA (G), TTT (F), CCA (P), and GTG (V). However, in reptiles, the most frequently used codons amounted to 26 including T-11, A-10, G-3, and A-2 in which four A-ending codons were over-represented, viz. GCA (A), AGA (R), GGA (G), and CCA (P). Lastly, in mammals, the most frequently used codons totaled 29, including the codons ending with A-10, T-9, C-6, and G-4, whereas the over-represented codons include only CGA, GGA, and GTG encoding the amino acids arginine (R), glycine (G), and valine (V), respectively. 

### 3.3. Analysis of PR2 Plot 

**Figure 4:**
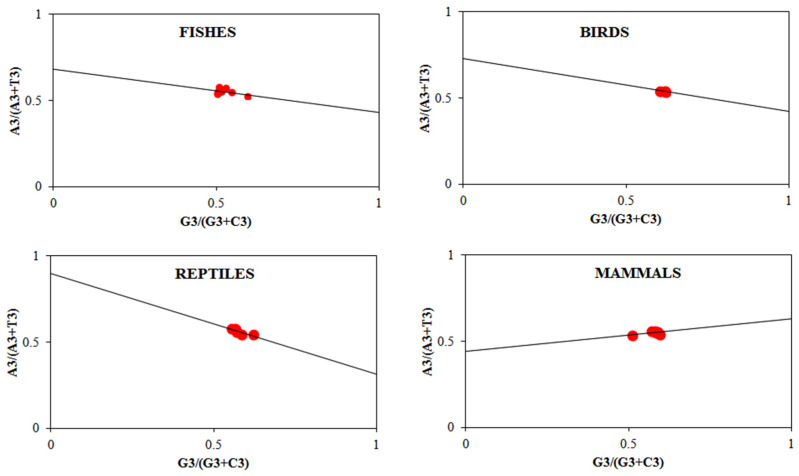
Parity plot analysis of *Foxp2* gene among different species of fishes, reptiles, birds, and mammals showing mean values of both AT bias and GC bias were greater than 0.5, which indicates the preferential usage of purine over pyrimidine at the third-position codon. We have plotted the values of GC bias [G3/(G3 + C3)] along the *X*-axis and the values of AT [A3/(A3 + T3)] bias along the *Y*-axis.

Parity rule 2 plot (PR2) analysis for the *FoxP2* gene among different species of the studied organisms showed that the mean value of both GC bias [G3/(G3 + C3)] and AT bias [A3/(A3 + T3)] was greater than 0.5 (Figure 4), which revealed that at the third position, purine was preferred over pyrimidine (G over C and A over T). If the codon bias is affected by the composition of the nucleotide, the third position between G3 and C3 along with A3 and T3 should have identical distributions, and this strand-specific rule is primarily irrespective of the G + C contents [21]. Therefore, the asymmetry between purine (GA) and pyrimidine (CT) indicates that aside from the nucleotide composition, other factors such as natural selection might influence the codon bias for the *FoxP2* gene in fishes, birds, reptiles, and mammals [47].

### 3.4. Analysis of Neutrality Plot

**Figure 5:**
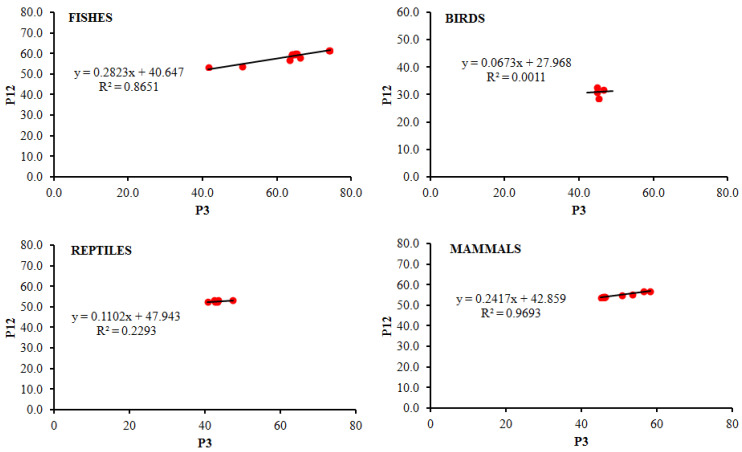
Neutrality plot (P12 versus P3) in the coding sequence of *Foxp2* gene among different species of fishes, birds, reptiles, and mammals. The values of P3 were plotted on *X*-axis and P12 (the average of P1 and P2) was plotted on *Y*-axis. Black line is the linear regression of P12 versus P3.

The neutrality plot depicts the influence of natural selection and mutation pressure on the codon bias of the gene by analyzing the correlation between P12 (the average of GC contents at positions P1 and P2) and P3 codon positions. The correlation coefficient (Figure 5) showed that a significant correlation between P12 and P3 for the *FoxP2* gene among fishes (r = 0.830, *p* < 0.01), birds (r = 1.000, *p* < 0.01), and mammals (r = 0.919, *p* < 0.01), excluding reptiles (r = 0.232), indicated directional mutation pressure acting on all codon positions. Moreover, slopes of regression lines for fishes, reptiles, birds, and mammals were 0.2823, 0.1102, 0.0673, and 0.2417, respectively, i.e., close to zero, suggesting the role of natural selection was higher than mutation pressure in influencing the CUB for the *FoxP2* gene among fishes, reptiles, birds, and mammals.

### 3.5. Correspondence Analysis

**Figure 6:**
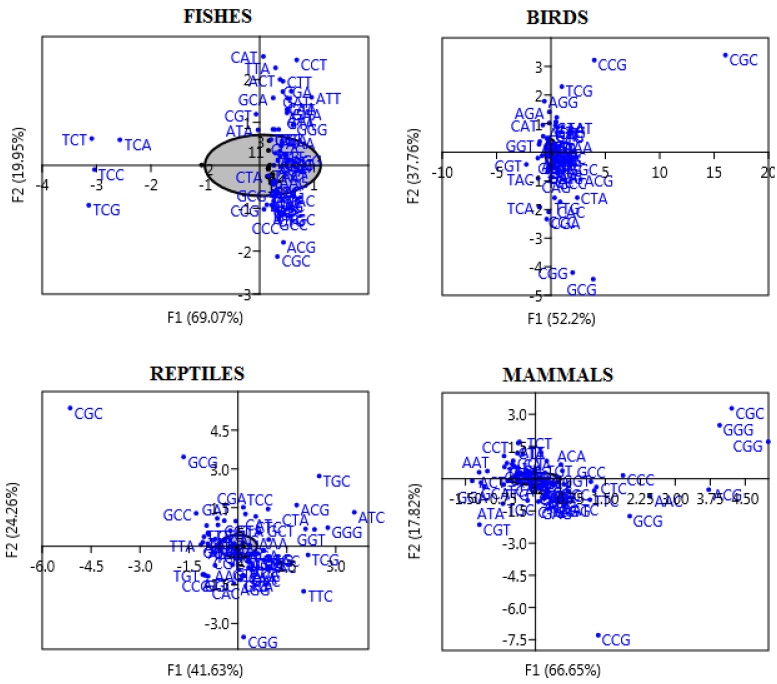
The correspondence analysis of RSCU values for *Foxp2* gene in fishes, birds, reptiles, and mammals. In the plot, each of the points indicate the variation of genes corresponding to the coordinate of first (*f1*) and second (*f2*) axes of variation.

The correspondence analysis (CoA) depicts the extent of genes and their respective codons, unveiling major effects on CUB [53]. The CoA analysis on RSCU values of 59 synonymous codons in this study (Figure 6) showed that the plot of each organism differs for the *FoxP2* gene, suggesting variation in the codon usage patterns. The major axis i.e., the first axis (*f1*) accounts for wide variations while the second axis (*f2*) accounts for narrow variation within the *FoxP2* gene of fishes, birds, reptiles, and mammals. However, a majority of the codons were confined closer to the axis around the center of the plot, indicating underlying mutation bias on the composition of the nucleobase might affect the codon usage of the *FoxP2* gene [54].

### 3.6. Analysis of Amino Acid Composition and Protein Properties

**Figure 7:**
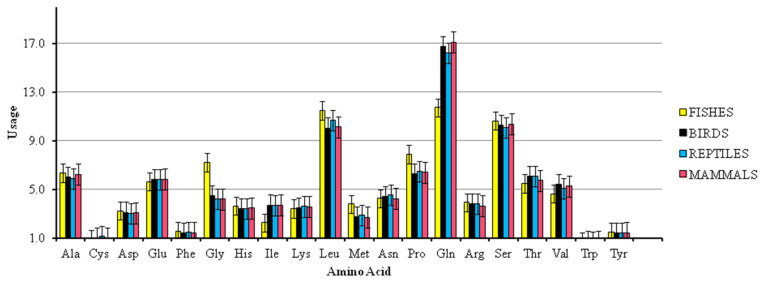
Overall frequency of amino acids used in the *Foxp2* gene among various species of fishes, reptiles, birds, and mammals. The amino acids glutamine (Gln), leucine (Leu), and serine (Ser) were predominant over other amino acids.

The amino acid compositions in the CDS of the *FoxP2* gene among the various species of fishes, birds, reptiles, and mammals were calculated. The overall frequency of each of the amino acids in *FoxP2* (Figure 7) revealed that Leucine, Glutamine, and Serine were predominant over other amino acids. The aromatic property of the amino acids (Phenylalanine, Tyrosine, and Tryptophan) present in the transcribed *FoxP2* gene product (AROMATICITY) was 0.04 ± 0.001 (mean ± SD) while the property of hydrophobicity (GRAVY) was 0.70 ± 0.001 (mean ± SD). The GRAVY score value was negative, suggesting soluble properties of the protein product.

### 3.7. Phylogenetic Analysis

**Figure 8:**
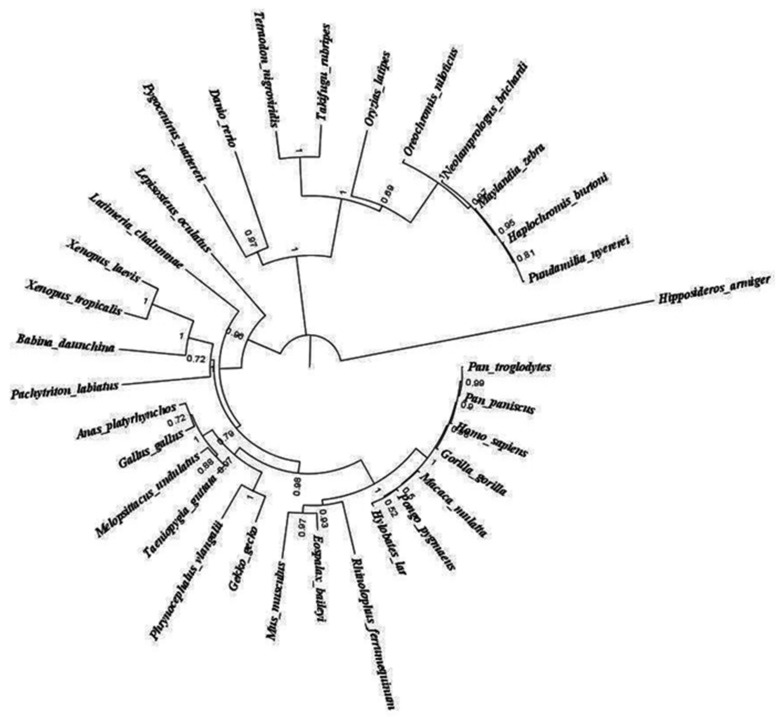
Phylogenetic analysis based on the coding sequence of *Foxp2* gene among fishes, birds, reptiles, and mammals (33 species). The tree was constructed using bootstrap analysis of 1000 replicates. Evolutionary analyses were conducted in MEGA6.

The neighbor-joining tree shows the same relationships between the *FoxP2* genes obtained from different species (Figure 8). Two major clades were observed. Among the species *Hipposideros armiger*, *FoxP2* genes have diverged from the rest of the mammalian *FoxP2* gene sequence.

## 4. Discussion

The current study highlights the pattern of codon usage of the *FoxP2* gene among fishes, reptiles, birds, and mammals. The study of the CUB of a gene is an important application in evolutionary biology and has been found in diverse groups of organisms, from unicellular prokaryotes to multicellular eukaryotes. The ’mutation-selection drift’ theory has been employed to depict the origin of codon usage bias (CUB) of a gene [55,56]. The theory explains that evolutionary forces such as the selection of compositional constraints, mutation pressure, along with genetic drift in a population might play an effective role in the usage of codon bias [57]. Earlier studies have reported that genes within a species exhibit similar patterns of codon usage [58,59]. 

Nucleotide composition plays an important role in influencing codon usage in genes as well as genomes. In our study of the *FoxP2* gene, we found GC and P3 content was higher than 50% in fishes, while in reptiles, birds, and mammals, GC content was lower than 50%. Earlier reports suggested that genes with high GC content provide more targets for methylation [60]. High GC content might assist more complex gene regulation [61]. Since GC and P3 contents were high in the *FoxP2* gene in fishes, they are more susceptible to mutation. This can be further supported by the neutrality plot, where mutational pressure is the highest in fish.

In the *FoxP2* gene, the average ENC values in different species of fishes, reptiles, birds, and mammals were more than 50, which indicates CUB was low. Similar results were reported in the mitochondrial ND2 gene among fishes, birds, and mammals where the average ENC value was greater than 50, thereby supporting our results [62]. Low CUB might be beneficial for efficient replication in each cell, with potentially different codon preferences [63]. 

Two evolutionary forces such as natural selection and mutation pressure influenced CUB in the *FoxP2* gene. We observed that the slope of the regression line was close to zero in all fishes, reptiles, birds, and mammals, which indicates the dominant role of natural selection rather than mutation pressure. Based on the regression coefficient, the role of mutation pressure was highest in fishes compared to reptiles, birds, and mammals. In ATP genes, the role of natural selection was higher than mutation pressure, and based on the regression coefficient, the role of mutation pressure was highest in fishes in compared to birds and mammals, supporting our results [64]. 

In summary, we found that codon usage bias in the coding sequences of the *FoxP2* gene was relatively weak and influenced by natural selection along with nucleotide composition under mutation pressure. However, natural selection played a major role in comparison to mutation pressure in shaping the codon usage pattern, depicting weaker CUB. The over-represented codons (RSCU > 1.6, Table S3) in the coding sequences of the *FoxP2* gene in fishes were AGA (R), CAG (Q), GGC (G), ATC (Ile), CTG (L), AGC (S), and GTG (V) while in birds the over-represented codons were GCA (A), AGA (R), GGA (G), TTT (F), CCA (P), and GTG (V). Similarly, the over-represented codons of *FoxP2* genes in reptiles were GCA (A), AGA (R), GGA (G), and CCA (P), but in mammals, the over-represented codons of *FoxP2* were CGA (R), GGA (G), and GTG (V). Moreover, it was observed that the most frequently used codons ended with C or G in fishes, whereas the codons ending with T or A were most frequently used in the genomes of birds, reptiles, and mammals. This study will help us to understand the CUB of the *FoxP2* gene, which could further be used to explore their biology, particularly with regard to the mechanisms of communication among animals.

## 5. Conclusions

The codon usage bias in the *FoxP2* gene among different species of fishes, birds, reptiles and mammals revealed that the most preferred codon used by fishes were either C or G-ending codons while in birds, reptiles and mammals the mostly used preferred codons were either ending with A or T. Apart from the nucleotide composition, natural selection and mutation pressure might influence the CUB. The ENC observed/ENC expected ratio demonstrated that mutation pressure influences *FoxP2* codon usage patterns. Natural selection plays a major role over mutation pressure in the codon usage of *FoxP2* gene. Besides, nucleotide composition under the influence of mutation bias also contributes the codon usage of FoxP2 gene and nature has favored nearly all the synonymous codons for encoding the corresponding amino acid.

## Figures and Tables

**Table 1:** Characteristics of *FoxP2* gene among different groups of animals.

	Fish	Birds	Reptiles	Mammals
Length_cds_	2228 ± 101.57	2118 ± 28.14	2153 ± 42.20	2134 ± 36.45
GCcds	55.8 ± 4.05	47.7 ± 0.48	46.8 ± 0.70	49.2 ± 1.98
P1	61.2 ± 1.64	59.2 ± 0.14	58.7 ± 0.67	60.2 ± 0.45
P2	43.0 ± 2.33	38.4 ± 0.68	38.2 ± 0.20	38.3 ± 0.72
P3	63.2 ± 8.99	45.3 ± 0.86	43.4 ± 2.05	49.0 ± 4.86
P12	52.1 ± 1.92	31.6 ± 0.07	48.4 ± 0.28	49.3 ± 0.57
ENC	52.22 ± 3.47	53.05 ± 1.95	54.65 ± 1.23	54.63 ± 1.24
GRAVY	−0.62 ± 0.04	−0.74 ± 0.01	−0.72 ± 0.04	−0.74 ± 0.04
AROMATICITY	0.04 ± 0.001	0.01 ± 0.001	0.04 ± 0.001	0.04 ± 0.002

Length_cds_: Length of the coding sequences; GCcds: GC contents of the coding sequences; P1: GC content at the 1st codon position; P2: GC content at the 2nd codon position; P3: GC content at the third codon position; P12: The average of P1 and P2; ENC: Effective number of codons; GRAVY: Hydrophobicity of amino acid; AROMATICITY: Aromatic properties of amino acid.

**Table 2:** Correlation analysis between overall nucleotide and the corresponding nucleotide at the third codon position.

FISH	A3%	T3%	G3%	C3%	GC3%
A%	r = 0.966 **	r = 0.912 **	r = −0.931 **	r = −0.946 **	r = −0.945 **
T%	r = 0.979 **	r = 0.989 **	r = −0.971 **	r = −0.993 **	r = −0.990 **
G%	r = −0.952 **	r = −0.892 **	r = 0.915 **	r = 0.929 **	r = 0.928 **
C%	r = −0.984 **	r = −0.988 **	r = 0.974 **	r = 0.993 **	r = 0.992 **
GC%	r = −0.986 **	r = −0.958 **	r = 0.962 **	r = 0.980 **	r = 0.978 **
**BIRDS**	**A3%**	**T3%**	**G3%**	**C3%**	**GC3%**
A%	r = 0.980 *	r = 0.898	**r = −0.893**	r = −0.983 *	r = −0.983 *
T%	r = 0.649	r = 0.967 *	**r = −0.816**	**r = −0.887**	**r = −0.887**
G%	**r = −0.835**	**r = −0.865**	r = 0.700 *	r = 0.941	r = 0.929
C%	**r = −0.881**	**r = −0.981**	r = 0.908	r = 0.994 **	r = 0.994 **
GC%	**r = −0.915**	r = −0.963 *	r = 0.902	r = 1.000 **	r = 0.999 **
**REPTILES**	**A3%**	**T3%**	**G3%**	**C3%**	**GC3%**
A%	r = 0.967 **	r = 0.299	**r = −0.879**	**r = −0.452**	r = −0.957 **
T%	r = −0.389 **	r = 0.553	r = 0.290	**r = −0.285**	r = 0.056
G%	r = −0.944 **	r = 0.222	r = 0.958 **	r = −0.111 **	r = 0.684
C%	r = 0.081	r = −0.931 **	**r = −0.166**	r = 0.865 *	r = 0.384
GC%	r = −0.827 *	**r = −0.549**	r = 0.767	r = 0.605	r = 0.961 **
**MAMMALS**	**A_3_%**	**T_3_%**	**G_3_%**	**C_3_%**	**GC_3_%**
A%	r = 0.987 **	r = 0.956 **	r = −0.812 **	r = −0.968 **	r = −0.983 **
T%	r = 0.954 **	r = 0.998 **	r = −0.913 **	r = −0.893 **	r = −0.983 **
G%	r = −0.968 **	r = −0.984 **	r = 0.950 **	r = 0.870 **	r = 0.985
C%	r = −0.972 **	r = −0.931 **	r = 0.739 **	r = 0.990 **	r = 0.964 **
GC%	r = −0.989 **	r = −0.980 **	r = 0.858 **	r = 0.953 **	r = 0.995 **

** *p* < 0.01, * *p* < 0.05, Bold: negative correlation.

**Table 3:** Correlation analysis (Spearman rank) between overall ENC and P1, P2, P3, and P12 values of *FoxP2* gene among fish, birds, reptiles, and mammals.

ENC	P1	P2	P3	P12
BIRDS	−0.200	0.800	1.000 **	0.949
FISH	−0.742 **	−0.505	−0.938 **	−0.754 **
REPTILES	0.290	−0.493	0.543	0.638
MAMMALS	0.251	−0.257	0.305	−0.046

Here, ** *p* < 0.01.

## Data Availability

Available data are presented in the manuscript and its Supplementary Materials.

## Supplementary Materials

The following are available online at https://www.mdpi.com/article/10.3390/biology10111078/s1, Table S1: The accession number of the coding sequences of *FoxP2* gene among different species of fishes, birds, reptiles, and mammals. Table S2: Composition of the nucleotide and ENC value along with the mean and standard deviation in the CDS of FoxP2 gene among different species of fish, birds, reptiles, and mammals. Table S3: The overall relative synonymous codon usage patterns (RSCU) in the coding sequences of *FoxP2* gene among fishes, birds, reptiles, and mammals.
