# Supplementary material for: Analysis of Codon Usage of Speech Gene FoxP2 among Animals
Source: Biology (Basel). 2021 Oct 21;10(11):1078. doi: 10.3390/biology10111078 (PMC8614651; doi:10.3390/biology10111078)
Supplement: Supplementary file 1 [file biology-10-01078-s001.zip › biology-1378170-supplementary.pdf]

Table S1. The accession number of the coding sequences of *Foxp2* gene among different species of fishes, birds, reptiles and mammals

Animals	Species Name	Accession Number
Fishes	*Oryzias latipes*	EU143691.1
	Pygocentrus nattereri	AB302880.1
	Latimeria chalumnae	BK008622.1
	Maylandia zebra	BK008621.1
	Haplochromis burtoni	BK008620.1
	Pundamilia nyererei	BK008619.1
	Neolamprologus brichardi	BK008618.1
	Oreochromis niloticus	BK008617.1
	Tetraodon nigroviridis	BK008615.1
	Takifugu rubripes	BK008614.1
	Lepisosteus oculatus	BK008612.1
	Danio rerio	DQ061052.1
Birds	*Taeniopygia guttata*	AY395709.1
	Melopsittacus undulatus	AY466101.1
	Gallus gallus	JN677532.1
	Anas platyrhynchos	XM_021279765.2
Reptiles	*Xenopus tropicalis*	BC135996.1
	Xenopus laevis	BC170268.1
	Gekko gecko	JX543515.1
	Phrynocephalus vlangalii	JX543514.1
	Babina daunchina	JX543513.1
	Pachytriton labiatus	JX543512.1
Mammals	*Homo sapiens*	NM_014491
	Eospalax baileyi	JQ929758.1
	Pongo pygmaeus	AH011319
	Gorilla gorilla	AH011318
	Hylobates lar	AH011317
	Pan troglodytes	AH011316
	Mus musculus	AY079003
	Pan paniscus	AY143179.1
	Rhinolophus ferrumequinum	KJ645605.1
	Macaca mulatta	AF512950.1
	Hipposideros armiger	JN696404.1

Table S2. Composition of the nucleotide and ENC value along with the mean and standard deviation in the CDS of *FoxP2* gene among different species of fish, birds, reptiles and mammals

FISH	A %	T %	G %	C %	A3 %	T3 %	G3 %	C3 %	GC %	AT %	GC1 %	GC2 %	GC3 %	AT3 %	ENC
Oryzias latipes	23.7	15.8	28.6	32.0	14.2	11.7	37.5	36.6	60.5	39.5	62.6	44.9	74.1	25.9	44.9
Pygocentrus nattereri	26.8	17.2	25.3	30.7	19.3	14.3	35.1	31.3	56.0	44.0	59.9	41.6	66.4	33.6	48.6
Latimeria chalumnae	31.7	21.7	21.5	25.1	30.6	27.7	24.9	16.8	46.6	53.4	59.8	38.3	41.6	58.4	54.9
Maylandia zebra	25.7	17.4	26.7	30.2	19.8	16.1	33.0	31.1	56.9	43.1	61.9	44.7	64.1	35.9	53.2
Haplochromis burtoni	25.7	17.4	26.7	30.2	19.8	16.1	33.0	31.1	56.9	43.1	62.1	44.5	64.1	35.9	54.0
Pundamilia nyererei	25.7	17.4	26.7	30.2	19.8	15.9	33.2	31.1	57.0	43.0	62.1	44.5	64.2	35.8	54.0
Neolamprologus brichardi	25.7	17.2	26.5	30.7	19.5	15.7	33.2	31.7	57.1	42.9	61.9	44.7	64.9	35.1	53.0
Oreochromis niloticus	25.5	17.2	26.7	30.6	19.3	15.7	33.0	32.0	57.3	42.7	62.1	44.8	65.0	35.0	53.2
Tetraodon nigroviridis	25.5	17.2	26.5	30.7	18.6	16.0	33.0	32.5	57.2	42.8	62.3	43.9	65.4	34.6	51.2
Takifugu rubripes	23.9	15.9	28.0	32.2	14.0	11.7	37.3	36.9	60.2	39.8	62.7	43.8	74.3	25.7	47.8
Lepisosteus oculatus	30.3	20.5	22.6	26.7	27.0	22.4	27.6	23.0	49.3	50.8	57.6	39.5	50.7	49.4	56.7
Danio rerio	28.3	17.2	24.2	30.2	21.1	15.4	32.3	31.3	54.4	45.6	59.2	40.7	63.5	36.5	55.2
Mean	26.5	17.7	25.8	30.0	20.3	16.5	32.8	30.4	55.8	44.2	61.2	43.0	63.2	36.8	52.2
SD	2.4	1.7	2.1	2.0	4.6	4.4	3.6	5.5	4.1	4.1	1.6	2.3	9.0	9.0	3.5
BIRDS	A %	T %	G %	C %	A3 %	T3 %	G3 %	C3 %	GC %	AT %	GC1 %	GC2 %	GC3 %	AT3 %	ENC
Taeniopygia guttata	31.8	20.8	22.3	25.2	29.9	25	27.7	17.0	47.4	52.6	59.3	38.2	44.8	55.2	53.4
Melopsittacus undulatus	31.7	21.0	22.2	25.1	29.6	26	27.7	16.9	47.3	52.7	59.2	38.0	44.7	55.4	50.3
Anas platyrhynchos	31.1	20.6	22.7	25.7	29.0	24	28.0	18.5	47.6	52.4	59.4	38.1	45.2	54.8	53.6
Gallus gallus	31.6	20.8	22.2	25.3	29.6	25	27.9	17.3	48.4	51.6	59.1	39.5	46.5	53.5	54.9
Mean	31.5	20.8	22.3	25.3	29.5	25.2	27.9	17.4	47.7	52.3	59.2	38.4	45.3	54.7	53.1
SD	0.32	0.19	0.23	0.27	0.34	0.57	0.15	0.73	0.48	0.48	0.14	0.68	0.86	0.86	1.95
REPTILES	A %	T %	G %	C %	A3 %	T3 %	G3 %	C3 %	GC %	AT %	GC1 %	GC2 %	GC3 %	AT3 %	ENC
Xenopus tropicalis	32.6	20.9	20.7	25.8	32.5	24.2	24.6	18.7	46.5	53.5	57.8	38.4	43.3	56.7	54.7
Xenopus laevis	33.2	20.9	20.4	25.5	34.2	25.0	23.1	17.7	45.9	54.1	58.6	38.3	40.7	59.3	53.5
Gekko gecko	32.2	21.4	21.4	25.1	31.1	26.1	25.1	17.7	46.5	53.5	58.3	38.2	42.8	57.2	53.1
Phrynocephalus vlangalii	32.2	20.9	21.8	25.1	30.6	25.9	27.1	16.4	46.9	53.1	59.4	37.8	43.5	56.5	54.8
Babina daunchina	32.8	20.4	20.4	26.3	33.4	24.2	23.5	18.9	46.8	53.2	59.7	38.2	42.4	57.6	55.3
Pachytriton labiatus	31.2	20.9	21.7	26.2	29.2	23.3	27.2	20.3	48.0	52.0	58.3	38.1	47.5	52.5	56.5
Mean	32.4	20.9	21.1	25.7	31.8	24.8	25.1	18.3	46.8	53.3	58.7	38.2	43.4	56.6	54.7
SD	0.70	0.30	0.63	0.53	1.87	1.08	1.75	1.34	0.70	0.70	0.73	0.22	2.24	2.23	1.23
MAMMALS	A %	T %	G %	C %	A3 %	T3 %	G3 %	C3 %	GC %	AT %	GC1 %	GC2 %	GC3 %	AT3 %	ENC
Homo sapiens	31.9	20.3	21.7	26.0	30.6	24.3	26.5	18.6	47.7	52.3	60.1	38	45.1	54.9	54.5
Eospalax baileyi	30.6	19.7	22.9	26.8	26.7	22.5	30.3	20.5	49.7	50.3	60.3	37.8	50.8	49.2	54.9
Pongo pygmaeus	31.6	20.4	21.9	26.1	29.6	24.2	27.0	19.2	48	52.0	59.9	38	46.2	53.8	54.4
Gorilla gorilla	31.7	20.4	21.8	26.0	30.1	24.5	26.8	18.6	47.8	52.2	59.9	38.1	45.4	54.6	54.2
Hylobates lar	31.7	20.3	21.8	26.2	30.1	24.1	26.6	19.2	47.9	52.1	59.9	38.1	45.8	54.2	54.6
Pan troglodytes	31.7	20.3	21.9	26.2	29.8	24.1	27.1	19.0	48	52.0	60.1	37.9	46	54.0	54.8
Mus musculus	30.4	19.1	23.2	27.3	26.0	20.6	31.0	22.4	50.5	49.5	60	38	53.4	46.6	56.8
Pan paniscus	31.8	20.3	21.8	26.1	30.1	24.1	26.9	18.8	47.9	52.1	60.1	37.9	45.7	54.3	54.7
Macaca mulatta	28.7	18.2	24.2	28.9	23.5	18.3	33.2	25.0	53.1	46.9	61	39.9	58.2	41.8	51.9
Rhinolophus ferrumequinum	31.6	20.3	21.9	26.2	29.7	24.2	27.1	19.0	48.1	51.9	60	38.04	46.2	53.9	56.2
Hipposideros armiger	28.7	18.9	23.3	29.1	23.3	20.3	28.7	27.7	52.4	47.6	61.2	39.6	56.4	43.6	53.9
Mean	31.0	19.8	22.4	26.8	28.1	22.8	28.3	20.7	49.2	50.8	60.2	38.3	49.0	51.0	54.6
SD	1.22	0.77	0.85	1.16	2.78	2.15	2.25	3.06	1.98	1.96	0.45	0.72	4.86	4.87	1.24

SD: Standard Deviation

TableS3a. Overall relative synonymous codon usage patterns (*RSCU*) in the coding sequences of *Foxp2* gene among fishes

Amino Acid		Codon	N	RSCU[a]	Amino Acid		Codon	N	RSCU[a]
Ala	A	GCA*	14	1.2	Leu	L	TTA	9	0.6
		GCC*	18	1.5			TTG	10	0.7
		GCG	4	0.4			CTA	7	0.5
		GCT	11	0.9			CTC*	18	1.3
Arg	R	CGT	3	0.7			CTG*	30	2.1
		CGC*	6	1.2			CTT	11	0.8
		CGA	3	0.5	Lys	K	AAA	10	0.8
		CGG	4	0.9			AAG*	15	1.2
		AGA*	8	1.7	Phe	F	TTT*	8	1.3
		AGG	5	1.0			TTC	4	0.7
Asn	N	AAC*	21	1.3	Pro	P	CCA*	18	1.2
		AAT	11	0.7			CCC*	22	1.5
Asp	D	GAT	7	0.6			CCG	9	0.6
		GAC*	17	1.4			CCT	9	0.6
Cys	C	TGC	3	0.9	Ser	S	TCA	12	0.9
		TGT*	4	1.1			TCC	13	1.0
Gln	Q	CAA	19	0.4			TCG	7	0.5
		CAG*	68	1.6			TCT	9	0.7
Glu	E	GAA	14	0.7			AGC*	24	1.8
		GAG*	28	1.3			AGT	14	1.0
Gly	G	GGA*	15	1.1	Thr	T	ACA	11	1.0
		GGC*	22	1.7			ACC*	14	1.4
		GGG	7	0.5			ACG	9	0.9
		GGT	9	0.7			ACT	7	0.7
His	H	CAT	6	0.5	Tyr	Y	TAC	5	1.0
		CAC*	21	1.5			TAT	6	1.0
Ile	I	ATA	4	0.7	Val	V	GTA	6	0.7
		ATC*	9	1.6			GTC*	10	1.1
		ATT	4	0.7			GTG*	15	1.8
							GTT	4	0.5

[a] mean values of *RSCU* based on the synonymous codon usage frequencies of Foxp2 gene; N: Total number of preferred codon; **RSCU*>1; yellow colored: Over represented codon

TableS3b. Overall relative synonymous codon usage patterns (*RSCU*) in the coding sequences of *Foxp2* gene among birds

Amino Acid	Codon	N	RSCU[a]	Amino Acid	Codon	N	RSCU[a]
Ala A	GCA*	24	2.3	Leu L	TTA*	14	1.2
	GCC	7	0.7		TTG	12	1.0
	GCG	1	0.1		CTA	5	0.4
	GCT	9	0.9		CTC	11	0.9
Arg R	CGT	5	1.0		CTG*	14	1.2
	CGC	1	0.1		CTT*	15	1.3
	CGA*	5	1.1	Lys K	AAA*	13	1.1
	CGG	3	0.6		AAG	11	0.9
	AGA*	8	1.9	Phe F	TTT*	8	1.6
	AGG*	6	1.3		TTC	2	0.4
Asn N	AAC	10	0.7	Pro P	CCA*	20	1.8
	AAT*	21	1.3		CCC	8	0.8
Asp D	GAT*	13	1.2		CCG	2	0.2
	GAC	9	0.8		CCT*	14	1.3
Cys C	TGC	2	0.6	Ser S	TCA	11	0.9
	TGT*	5	1.4		TCC	12	1.0
Gln Q	CAA	35	0.6		TCG	4	0.3
	CAG*	83	1.4		TCT*	14	1.2
Glu E	GAA*	26	1.3		AGC*	18	1.5
	GAG	15	0.7		AGT*	15	1.2
Gly G	GGA*	17	2.2	Thr T	ACA*	14	1.3
	GGC*	9	1.2		ACC	9	0.9
	GGG	2	0.3		ACG	3	0.3
	GGT	3	0.4		ACT*	16	1.5
His H	CAT*	15	1.3	Tyr Y	TAC	5	0.9
	CAC	9	0.7		TAT*	5	1.1
Ile I	ATA	9	1.0	Val V	GTA	7	0.7
	ATC	5	0.6		GTC	7	0.8
	ATT*	12	1.4		GTG*	16	1.7
					GTT	8	0.8

[a] mean values of *RSCU* based on the synonymous codon usage frequencies of Foxp2 gene; N: Total number of preferred codon; **RSCU*>1; yellow colored: Over represented codon

TableS3c. Overall relative synonymous codon usage patterns (*RSCU*) in the coding sequences of *Foxp2* gene among reptiles

Amino Acid	Codon	N	RSCU[a]	Amino Acid	Codon	N	RSCU[a]
Ala A	GCA*	21	2.0	Leu L	TTA*	15	1.2
	GCC	7	0.7		TTG	13	1.0
	GCG	3	0.2		CTA	8	0.6
	GCT*	12	1.1		CTC	9	0.7
Arg R	CGT	3	0.7		CTG*	14	1.1
	CGC	1	0.2		CTT*	18	1.5
	CGA*	7	1.4	Lys K	AAA*	15	1.2
	CGG	3	0.6		AAG	11	0.9
	AGA*	9	2.1	Phe F	TTT*	7	1.3
	AGG	5	1.0		TTC	4	0.7
Asn N	AAC	14	0.8	Pro P	CCA*	22	1.9
	AAT*	19	1.2		CCC	8	0.7
Asp D	GAT	11	1.0		CCG	2	0.2
	GAC	11	1.0		CCT*	13	1.2
Cys C	TGC	3	0.6	Ser S	TCA*	14	1.2
	TGT*	6	1.4		TCC	10	0.8
Gln Q	CAA	46	0.8		TCG	2	0.2
	CAG*	71	1.2		TCT*	14	1.2
Glu E	GAA*	26	1.3		AGC*	17	1.4
	GAG	16	0.8		AGT*	15	1.2
Gly G	GGA*	14	1.8	Thr T	ACA*	14	1.3
	GGC*	8	1.1		ACC	10	0.9
	GGG	4	0.5		ACG	3	0.3
	GGT	5	0.7		ACT*	17	1.5
His H	CAT*	13	1.1	Tyr Y	TAC	5	1.0
	CAC	12	1.0		TAT	5	1.0
Ile I	ATA	8	0.9	Val V	GTA	9	0.9
	ATC	6	0.7		GTC	9	1.0
	ATT*	12	1.4		GTG*	10	1.1
					GTT	9	1.0

[a] mean values of *RSCU* based on the synonymous codon usage frequencies of Foxp2 gene; N: Total number of preferred codon; **RSCU*>1; yellow colored: Over represented codon

TableS3d. Overall relative synonymous codon usage patterns (*RSCU*) in the coding sequences of *Foxp2* gene among mammals

Amino Acid	Codon	N	RSCU[a]	Amino Acid	Codon	N	RSCU[a]
Ala A	GCA*	17	1.5	Leu L	TTA*	14	1.2
	GCC*	14	1.2		TTG	9	0.8
	GCG	4	0.4		CTA	7	0.6
	GCT	10	0.9		CTC	12	1.0
Arg R	CGT	4	0.8		CTG*	15	1.2
	CGC	1	0.3		CTT*	15	1.3
	CGA*	7	1.7	Lys K	AAA*	15	1.2
	CGG	2	0.5		AAG	11	0.9
	AGA*	7	1.5	Phe F	TTT*	8	1.5
	AGG*	5	1.3		TTC	2	0.5
Asn N	AAC	9	0.6	Pro P	CCA*	15	1.3
	AAT*	21	1.4		CCC	9	0.8
Asp D	GAT	9	0.8		CCG	4	0.4
	GAC*	13	1.2		CCT*	17	1.5
Cys C	TGC	3	0.8	Ser S	TCA*	14	1.1
	TGT*	4	1.2		TCC	11	0.9
Gln Q	CAA	37	0.6		TCG	5	0.4
	CAG*	85	1.4		TCT	12	0.9
Glu E	GAA*	25	1.2		AGC*	17	1.4
	GAG	17	0.8		AGT*	16	1.3
Gly G	GGA*	16	2.1	Thr T	ACA*	13	1.2
	GGC*	9	1.2		ACC*	13	1.3
	GGG	3	0.4		ACG	2	0.2
	GGT	2	0.3		ACT*	13	1.3
His H	CAT*	13	1.1	Tyr Y	TAC*	6	1.1
	CAC	12	0.9		TAT	4	0.9
Ile I	ATA	9	1.0	Val V	GTA	7	0.8
	ATC	8	0.9		GTC	9	1.0
	ATT*	9	1.1		GTG*	16	1.7
					GTT	5	0.5

[a] mean values of *RSCU* based on the synonymous codon usage frequencies of Foxp2 gene; N: Total number of preferred codon; **RSCU*>1; yellow colored: Over represented codon
